# Soil-Transmitted Helminth Vaccines: Are We Getting Closer?

**DOI:** 10.3389/fimmu.2020.576748

**Published:** 2020-09-30

**Authors:** Ayat Zawawi, Kathryn J. Else

**Affiliations:** ^1^Department of Medical Laboratory Technology, Faculty of Applied Medical Sciences, King Abdulaziz University, Jeddah, Saudi Arabia; ^2^Manchester Academic Health Science Centre, Faculty of Biology, Medicine, and Health, School of Biological Sciences, Lydia Becker Institute of Immunology and Inflammation, University of Manchester, Manchester, United Kingdom

**Keywords:** helminth, hookworm, *Trichuris*, *Ascaris*, vaccine, vaccine-induced immunity

## Abstract

Parasitic helminths infect over one-fourth of the human population resulting in significant morbidity, and in some cases, death in endemic countries. Despite mass drug administration (MDA) to school-aged children and other control measures, helminth infections are spreading into new areas. Thus, there is a strong rationale for developing anthelminthic vaccines as cost-effective, long-term immunological control strategies, which, unlike MDA, are not haunted by the threat of emerging drug-resistant helminths nor limited by reinfection risk. Advances in vaccinology, immunology, and immunomics include the development of new tools that improve the safety, immunogenicity, and efficacy of vaccines; and some of these tools have been used in the development of helminth vaccines. The development of anthelminthic vaccines is fraught with difficulty. Multiple lifecycle stages exist each presenting stage-specific antigens. Further, helminth parasites are notorious for their ability to dampen down and regulate host immunity. One of the first significant challenges in developing any vaccine is identifying suitable candidate protective antigens. This review explores our current knowledge in lead antigen identification and reports on recent pre-clinical and clinical trials in the context of the soil-transmitted helminths *Trichuris*, the hookworms and *Ascaris*. Ultimately, a multivalent anthelminthic vaccine could become an essential tool for achieving the medium-to long-term goal of controlling, or even eliminating helminth infections.

## Introduction

Neglected tropical diseases (NTDs) remain a global public health issue. Their impact is especially felt in areas with poor hygiene and sanitation, and are related to extreme poverty and lack of health education (1). Some of the most “neglected” diseases are caused by soil-transmitted helminths (STHs), which together infect more than a quarter of the world's population (2). The four most prevalent STHs worldwide are the Hookworms *Necator americanus* and *Ancylostoma duodenale*, the roundworm *Ascaris lumbricoides, Strongyloides stercoralis*, together with the whipworm *Trichuris trichiura* (3). The highest intensity of infection for *A. lumbricoides* and *T. trichiura* is typically seen in school-aged children and adolescents, whilst in hookworm infections infection intensity tends to increase with age, plateauing in to adulthood. Those who travel to and from regions where STH infections are endemic are also at risk of getting infected (4, 5). Although little is known about *S. stercoralis* in comparison to *Ascaris, Trichuris* and hookworm despite prevalence estimates of up to 40% in tropical countries (6–9) there is a growing understanding of the immune response to infection (10, 11). However, a biochemical understanding of the parasite remains limited and thus, this review will focus on vaccines against the human hookworms (12), whipworm and roundworm (13).

The ability to control STH infections currently depends almost exclusively on mass anti-helminthic drug administrations (MDA) such as albendazole or mebendazole to at-risk populations (4, 5, 14, 15) in conjunction with education and improved sanitation including WASH initiatives (16, 17). However, post-treatment reinfection is common, especially for helminth species such as *Ascaris* and *Trichuris*, where the robust parasite eggs are nearly ubiquitous in the environment. Such extensive contamination of soil with parasite eggs limits the ability of MDA programs, global control and elimination efforts to interrupt the transmission cycle within a community (18). Moreover, the appearance of anthelmintic-resistant parasites threatening human drug treatment programmes (19, 20) has increased interest in developing vaccines, or a pan-anthelminthic vaccine to provide a cost-effective, long-term immunological method to control multiple helminth infections (21–23).

This review explores our current knowledge, prospects, and challenges for anti-human STHs vaccine design. Although of significant importance, the review does not cover veterinary vaccines which have been reviewed recently (24). We include the importance of understanding the underpinning immune responses required to eliminate STHs from their host, opportunities for antigen-presenting cell targeting of candidate antigens, and include recent pre-clinical and clinical trials in the context of *Ascaris, Trichuris* and the hookworms.

## Prospects and Challenges for Anti-STHs Vaccine Design

The development of efficient anti-STH vaccines is thought to represent a greater challenge compared to developing anti-bacterial or anti-viral vaccines. This is partly a result of the complex lifecycles of STHs, combined with an incomplete immunological knowledge of the host-parasite interactions and the immune mechanisms conferring protection (25). Moreover, STHs have complex genomes and proteomes (26–30). This complexity makes it difficult to identify antigenic targets for the development of an effective vaccine (31). Despite these obstacles, the development of vaccines against the STHs has progressed over the decades.

### Identifying Lead Antigens to Include in Vaccines

#### Crude Antigen Preparations

The earliest anthelmintic vaccines included attenuated or irradiation-killed parasites (32, 33). Since this time, extensive studies across many helminth spp have explored the immunogenicity of native molecules excreted and/or secreted by parasites (the so-called ES molecules) (34–37) and parasite-derived extracellular vesicles (EVs) in promoting resistance to infection (38–41). Excreted and secreted parasite molecules make excellent vaccine candidates as they sit at the host-parasite interface, playing critical roles in both the modulation of host immunity and in inducing Th2-skewed immune responses (35, 42).

#### Recombinant Proteins

Instead of targeting the immune response against a mixture of antigens, a particular antigen from the pathogen can be produced and expressed in a heterologous expression system to focus the immune response toward a specific antigen of the pathogen to prevent the infection (43, 44). Such an approach ensures that safer and more reliable vaccines are developed, with an example being the licensed recombinant hepatitis B vaccine (45). The key therefore to an effective recombinant vaccine is to identify that particular conserved antigen or combination of antigens secreted or extracted from the pathogen that can overcome the low protective immunity naturally generated by the infection (44, 46). However, recombinant vaccines often require an adjuvant and multiple immunizations to elicit a protective and long-lasting immune response (47). Moreover, the production of recombinant proteins using bacterial heterologous expressions can prove challenging. Thus, antigens in which proteolytic stability, higher production yield and post-translational modifications (e.g., phosphorylation and glycosylation) are needed, other expression systems such as yeast, insect, plant, or mammalian cells should be considered (44). Of the most successful helminth recombinant vaccines are the two against hookworm disease caused by Necator americanus, which consist of aspartic protease-1 (Na-APR-1) and glutathione-S-transferase-1 (Na-GST-1). These vaccines are currently in phase 1 of clinical trials (48–50).

#### DNA Based Vaccines

The availability of genome sequences opened up the prospect of the DNA vaccine approach to validate novel vaccine candidates individually or combined to improve vaccine elicitation of cell-mediated and mucosal immunity (51, 52). DNA vaccines are simple rings of DNA containing a gene encoding a specific vaccine antigen under the regulation of a promotor (53). DNA vaccine technology has been intensively used to develop vaccines against various pathogens (54, 55), cancer (56), and autoimmune disorders (57). For example, the human immunodeficiency virus-1 (both as a prophylactic and an immunotherapeutic vaccine) (58, 59), Zika virus (60), and Ebola virus vaccines (61) are currently in clinical trials. Comparatively, little progress has been made toward developing DNA vaccines against parasitic diseases, although several pre-clinical studies have shown promising results against hookworm infections (62, 63), malaria (64, 65), leishmaniasis (66, 67), and schistosomiasis (68, 69). Even though DNA vaccines offer several advantages when compared with recombinant vaccines, such as safety, improper protein folding and production cost, they have not been shown to be sufficient to induce a protective immune response (70). Therefore, DNA encapsulation, plasmid alterations, co-expressing cytokines and heterologous prime-boost approaches have been explored to enhance the immune responses induced by the DNA vaccine (71, 72).

#### Epitope-Based Vaccines

The advent of the genome era and the tremendous advances in immunological and bioinformatics tools have enabled the reverse vaccinology approach (RV) as a new effective strategy for lead antigen identification in vaccine development (73–75). One of the advantages of the RV approach is that every antigen encoded in the worm genome can be screened *in silico* to determine its ability to induce an immune response (31, 74). Thus, it can overcome some of the limitations of conventional methods of screening vaccine candidates (76–78). Antigen selection can also be carefully prescribed based on clear inclusion and exclusion criteria. For example, Zawawi et al. employed a systematic, multi-stage process to identify *Trichuris* epitope vaccine antigens based on the RV approach (79). The epitopes were identified from secreted, and surface-exposed proteins and any protein with any degree of homology to humans or mice were excluded to eliminate potential autoimmune reactions. Most of the identified vaccine antigens were stage-specific and had essential functions in the parasite biological process, associated with host-parasite interaction, parasite metabolism, development and fecundity (80).

#### The Search for a Pan-Anthelmintic Vaccine

Coinfections with two or multiple STHs are extremely common in sub-Saharan Africa and elsewhere in the developing countries (1, 81). The three STHs hookworm, *Ascaris* and *Trichuris* also share highly conserved antigens that are likely to have very similar biological functions (46). Thus, researchers have proposed a single pan-anthelmintic vaccine against the three major human STHs to generate strong, lasting immunity with minimal side effects (23). RV methodologies have much to offer in this context. Thus, with the availability of parasite genomes and bioinformatics tools to select out IgE-inducing epitopes *in silico*, the development of a pan-anthelmintic vaccine based on protective epitopes from cross-protective antigens may represent an exciting alternative to the use of whole antigens.

The choice of lead antigen also determines the biomechanical requirements of production, and the design of the laboratory and clinical trials (82–84). For example, vaccines based on native antigens, are known to generate significant immunity against many STHs in pre-clinical models (85–87). However, they have many manufactory limitations, such as high cost, time consumption, difficulty of purifying large quantities of worm antigens, low stability, shelf-life, shortage of *in vitro* methods for the culture of parasites and control over differences in batches to develop a commercially stable vaccine (88–90). Approaches such as recombinant (91), DNA (92), and epitope-based vaccines (93) overcome some of the limitations associated with native antigens. However, most vaccines against STHs remain in early-stage development or are undergoing pre-clinical evaluation.

### Adjuvants: Key Components in Modern Vaccinology

The effectiveness of lead antigens is often restrained by an inherent lack of immunostimulation. Therefore, efforts have focused on co-administering antigens with vaccine adjuvants as a key component in modern vaccinology, aiming to intensify the immune response and generate effective immunological memory (94). In addition, adjuvants are important in overcoming immune senescence seen in the elderly (95), and expanding the antibody repertoire generated (96). Adjuvants may also allow dose-sparing (97) and careful choice of adjuvants used provides the ability to guide the type of immune response generated (98, 99). The specific type of T helper response generated plays critical role in the efficacy of the protective immune response, as a Th1-type response is critical in developing vaccines against intracellular pathogens, whereas Th2-type responses are critical in developing vaccines against extracellular parasites (100).

Among the earliest adjuvants used in experimental antibody production were Freund's adjuvants (101, 102). However, as these adjuvants are associated with pain, inflammation, and tissue destruction they are no longer used and have been replaced by other adjuvants that can produce equal or superior antibody responses with less inflammation and tissue destructions (103).

The two adjuvants that are licensed for use in humans, aluminum salt and squalene oil-based emulsion (MF59), mainly promote Th2-biased immune responses (99, 104). Each adjuvant has its own immunological signature. For example, alum increased antibody titers, whereas MF59 induces strong antibody and IL-5 responses in mice (105). However, their mechanisms of action remain only partially understood.

Nanoparticulate vaccine adjuvants and delivery vehicles have also been incorporated into vaccine design to enhance the humoral and cellular immune responses (106, 107). Vaccine delivery systems include synthetic nano- and micro-particles, immunostimulatory complexes, liposomes, virosome, and virus-like particles (VLP) (108). These approaches promote the uptake of antigens by different antigen-presenting cells, promote their migration and maturation from the site of vaccine uptake and protect antigens from degradation (106, 109, 110). VLPs, for example, possess the immunostimulatory and self-adjuvanting properties of natural viruses but do not contain genetic material (111, 112). In addition, they are very stable and can withstand adverse environments, such as those with acidic pH, making VLPs an attractive carrier for mucosally administered vaccines (113). RTS, S (Mosquirix) was the first licensed VLP-based vaccine generated against parasitic disease (114). It is composed of three tandem repeat (R) and cell (T) epitopes from the circumsporozoite protein of the *P. falciparum* malaria parasite, which are displayed on hepatitis B surface particles (HBs-Ag) (S), co-expressed in *Saccharomyces cerevisiae* (S) and reconstituted with an AS01 adjuvant (115). The development of vaccines against *Toxoplasma gondii* (116), *T*. spiralis (117), *Clonorchis sinensis* vaccine (118), and *T. trichiura* (79) have all embraced VLP technology.

Antigens adjuvanted with toll-like receptor (TLR) agonist such as monophosphoryl lipid A (MPLA) and Glucopyransoyl lipid A (GLA) also enhance immune responses by inducing high antibody titres, driving the production of Th1 cytokines and rapid immune responses (119, 120). The TLR4 agonists are approved for use in human vaccines and are being studied in leishmaniasis (121), schistosomiasis (122), tuberculosis (123), and influenza vaccines (124). The combination of vaccine antigen with different TLR agonists has also been studied in various formulations such as microparticles, nanoparticles, and lipid emulsions to enhance the immune responses induced by the vaccine (125, 126). For example, mice immunized with a yellow fever vaccine containing antigens combined with TLR4 and TLR7 agonists and conjugated to synthetic nanoparticles, induced synergistic antigen-specific neutralizing antibodies compared to immunization with antigens coupled with a single TLR ligand (127). Further, an HIV vaccine conjugated with a TLR5 agonist combined to a nanoparticle synthesized from a synthetic poly lactic-*co*-glycolic acid (PLGA) showed increased immunogenicity in a preclinical mouse model and reduced the immunogenic dose of the vaccine candidate (128). Moreover, the formulation of a TLR4 ligand contained within a PLGA-based nanoparticle plus a malaria proteins (Pfs25) noted for its poor immunogenicity, improved vaccine induced-immunity (129).

Given the range of adjuvants available it is recommended to test several adjuvants or immunostimulants with each potential vaccine antigen at the pre-clinical stage of development in order to optimize the quality of the immune response and to generate effective protective immunity.

### Challenges to Vaccine Development

Having identified a lead antigen and selected an appropriate adjuvant, other hurdles remain to be overcome in the development of an anti-helminth vaccine. These include avoiding unwanted side effects, choosing a suitable animal model for testing immunogenicity and protective immunity, the immunization schedule, and the administration route to shape the immune response induced by the vaccine (82, 83).

Beyond the laboratory, other obstacles exist, including geopolitical barriers, unreliable pharmaceutical manufacturer markets, and low industry interest. Further, the rising anti-vaccine movement in the US and elsewhere have had a significant effect on STHs vaccine development (130). Thus, the development of new vaccines is a complex and multidisciplinary task that requires an understanding of host-pathogen interactions, epidemiology, and manufacturing parameters (131). Most importantly, vaccine researchers must have an understanding of the immune mechanisms involved in diseases and protection in order to select appropriate antigenic targets and delivery systems to shape the immune response induced by the vaccine (82, 83).

## Immune Responses to Hookworm, *Ascaris*, and *Trichuris* Infections

### Th2 Immune Responses Are Center Stage in Acquired Immunity to Infection

There is an abundance of literature documenting the need for strong Th2 biased immune responses to generate protective immune responses to STHs in animal models (132, 133). This is also clear in humans, where immunity to the human hookworms, *Ancylostoma duodenale* and *Necator americanus*, the roundworm *A. lumbricoides*, and the whipworm *T. trichiura* require the generation of strong Th2 immune responses and the production of Th2-associated cytokines including interleukin (IL)-4, IL-5, IL-6, IL-9, and IL-13 (134–136).

Infection of man with *N. americanus* and *T. trichiura* induces a mixed Th1/Th2 response characterized by the up-regulation of IFN-γ (Th1) and a strong Th2 response. As worm burdens decrease with age, or in the context of resistance to reinfection post drug treatment, so parasite-specific IgE levels increase accompanied by Th2 cytokines such as IL-5, IL-9, and IL-13 (134–138). The immune response to *A. lumbricoides* infection in 12–17 year old hosts living in endemic communities is more Th2 biased (IL-4 and IL-5) with no detectable production of IFN-γ in response to *Ascaris* antigens (139–141). Turner et al. (142) divided his study population of *Ascaris* infected individuals in to two age cohorts, 4–11 year olds and 12–36 year olds. This study concurred with Cooper et al. (139), reporting significant negative correlations between the Th2 cytokines IL-9 and IL-13 and infection intensity, but only in the older (over 12) age cohort. Thus, children below the age of 12 showed no inverse correlation between Th2 cytokines and intensity of infection (134, 142), suggesting that protection is conferred only after decades of exposure.

Collectively, therefore, there is strong evidence from both animal models and human field studies that any anti-STH vaccine should aspire to promote Th2 immunity.

### Identifying the Th2-Controlled Effector Mechanisms

Exactly how Th2 cytokines culminate in worm expulsion has been debated at length for many years, with our evidence base largely accruing from mouse models where the mechanism of action is best unpicked. Peripheral blood eosinophilia is a hallmark feature of the immune response to helminth infections (143–145). However, is it not clear whether eosinophils kill parasitic worms. IL-5 and eosinophils appear not to be essential for *Trichuris* and hookworm expulsion, as no difference in worm expulsion was observed following infection of IL-5 knockout mice (146, 147). In contrast, early studies demonstrated that eosinophils can kill infected larval stages of most helminth species investigated *in vitro* in the presence of specific antibodies or complement (148–150). A recent study also showed that eosinophils were recruited to the site of infection by immune serum activated macrophages, leading to the immobilization of migrating *A. suum* larvae (151).

Several studies have also highlighted a variety of Th2 regulated immune-mediated mechanisms associated with *Trichuris* expulsion including epithelial cell turn over (152, 153), increased muscle hyper-contractility (154), goblet cell hyperplasia and production of mucins (155), with specific Th2 cytokines identified as key. For example, several studies have provided evidence that IL-13, not IL-4 plays a critical role in increasing epithelial cell turnover and in the production of mucins to promote *Trichuris* worm expulsion (153, 156–159). IL-9 has been shown to be important in the stimulation of intestinal smooth muscle contractility, which drives *T. muris* expulsion (154, 160). In keeping with this, impaired IL-9 expression often results in chronic helminth infection (161).

Our understanding of mechanisms of immunity to hookworm and roundworm is less well-defined, largely due to the lack of robust mouse models. However, similar Th2-controlled effector mechanisms have been put forward for hookworm (12). For *Ascaris*, liver-stage immunity during the migratory stages of infection has been associated with oxidative phosphorylation and the production of reactive oxygen species (162), the lung-stage with an eosinophilia (163, 164), and expulsion from the gut with increased muscle contractility (165).

The role of the B cells and antibody in helminth infection remains unclear, with roles embracing both antibody production and antibody-independent cellular regulation. Thus, B-cells may play roles in stimulating the generation and/or polarization of T-cell responses by either cytokine secretion or antigen presentation in addition to their more widely appreciated role in antibody production (166, 167). In mouse models, both antibody-dependent and independent roles for the B cell have been proposed. Using a B-cell depletion strategy in a mouse model, a recent study suggested that the development of a Th2 type immune response to *Trichuris* infection is dependent on the host's genetic background and is independent of antibodies (168). Indeed in the context of *Trichuris* infections, evidence points toward antibody-independent worm expulsion mechanisms (169, 170). However, for other rodent helminths, roles for antibody have been well-documented (171, 172). For example, parasite-specific IgG1 is thought to play a role in immunity to the rodent hookworm *Heligmosomoides polygyrus* (173).

In humans, CD11c+ B cells have been shown to be the main IL-10 producers in Indonesian-STH-infected individuals compared to Europeans and Indonesians not exposed to helminths, inferring a regulatory function (174). The robust production of total and parasite-specific IgG1 and IgG4 have been associated with age, but may simply reflect the intensity of helminth infection (85, 134, 175). For example, children with repeatedly heavy infections with *A. lumbricoides* produced significantly higher levels of *A. lumbricoides*-specific (IgGl, IgG4, and IgE) compared to the repeatedly lightly infected children (140, 176). King et al. also suggested that antibody responses may not predict future levels of infection or confer protection from current infection or re-infection with *A. lumbricoides* but may only reflect infection intensity (177). In contrast, *Trichuris*-specific IgE has been shown to be negatively correlated with infection intensity and positively correlated with age, suggesting that IgE is associated with protection (134, 136). Total levels of IgE have also been correlated with the activation and degranulation of mast cells, basophils, and eosinophils (178). Further, negative associations between hookworm-specific intestinal and serum IgA and hookworm infection were observed in humans and the hamster model of *A. ceylanicum* hookworm infection, suggesting that antibodies may act in concert with other components of the mucosal and systemic immune response to promote protective immunity against hookworm infection (179–181). Overall, data supports the view that B cells are important in immunity to STHs, but the precise mechanisms by which B-cells and antibody support protective immunity remains only partially understood.

### Regulation of Host Immunity by STHs—A Challenge for Vaccine Development

Experimental model systems, both rodent and human, have demonstrated that helminth infections regulate host immunity, dampening pathology at the expense of efficient worm expulsion. Thus, helminth-derived products have been shown to suppress of Th1/Th17 responses, with suppression associated with the production of IL-10, IL-22 and transforming growth factor-β (182, 183). Peripheral blood from hookworm-infected individuals also show higher levels of circulating regulatory T cells expressing CTLA-4, GITR, IL-10, TGF-β, and IL-17 than healthy non-infected donors (184). In the context of animal models, mice deficient in IL-10 are susceptible to *T. muris* infection characterized by elevated levels of IFN-γ and TNF-α, and fatal intestinal pathology (185). Furthermore, depletion of regulatory T-cells during *T. muris* infection enables worm expulsion at the expense of increased intestinal inflammation (186). In the context of hookworm, and using a mouse model of colitis, *A. caninum* ES products were shown to suppress colitis. The suppression was associated with potent induction of IL-4 and IL-10 by CD4+ T cells in the draining lymph nodes and the colon together with the recruitment of alternatively activated (M2) macrophages and eosinophils to the site of ES administration (187).

Helminth induced immune regulation has been embraced by “worm therapy” advocates (182, 188, 189), and immune regulation by helminths is important in protecting the infected host from potentially life-threatening immunopathology (190, 191). However, the inherent dampening of the immune response associated with chronic worm infections represents a significant challenge in STH vaccine research as well as potentially compromising immunity to other vaccines and influencing the outcome of infection with co-infecting pathogens (192).

## Understanding How Vaccines Elicit Immunity: Can We Overcome the Challenges?

Vaccination is one of the greatest advances in global health; however, most successful vaccines have been made empirically. Despite a reasonable body of literature from animal models, describing how Th2 immune responses confer protection against STHs in a primary infection, there is very little data regarding the mechanism(s) of vaccine-driven immunity, and this represents a significant gap in our knowledge. Thus, although strong evidence exists to support the need for a vaccine to promote Th2 immune response against STHs in the context of potently regulated environments, how the Th2 immune response culminates in worm expulsion is unknown and may well differ to the Th2 controlled effector mechanisms at play in immunity to a primary infection. Further, we still have little insight into the mechanisms by which vaccines trigger Th1 or Th2 biased immune responses, strong B cell responses or long-lived memory T-cell responses, despite formation of T-cell memory being critical to protection against infectious diseases. Antigen delivery to the right antigen-presenting cells is critically important for the quality of the T-cell response, yet targeting of specific populations of antigen-presenting cells is often a neglected aspect in the design of vaccines.

There is a substantial evidence base to support the principle of dendritic targeting in vaccine development (193), although it has not been applied to many vaccines against STHs. Incorporating monoclonal antibodies that recognize, for example, dendritic cell surface molecules into delivery platforms, offers the prospect of direct delivery of antigen to specific antigen-presenting cell subsets *in vivo* (194–196), thus taking control of the quality of the subsequent vaccine-driven immune response (197). For example, delivery of antigen to CD8+ dendritic cells via Clec9A has been shown to promote CD4 T-cell responses and efficient development of T-follicular helper cells, important in antibody production (198). Interestingly, B-cells have been shown to be the dominant antigen-presenting cell-activating naïve CD4+ T-cells in response to virus-like particles (199), highlighting the importance of understanding how vaccines are presented *in vivo* as a prerequisite to developing antigen-presenting cell-targeting strategies.

## Soil-Transmitted Helminth Vaccines: Are We Getting Closer?

### Experimental Hookworms Vaccine Candidates

Hookworms (*Necator* americanus, *Ancylostoma duodenale*, and *Ancylostoma ceylanicum*) infect around 500 million people worldwide and are of significant concern due to their voracious blood-feeding (1). *N. americanus* and *A. duodenale* infect humans whereas *A. ceylanicum* and *A. caninum* are zoonotic and rarely infect humans. Hookworms can live for years in the host's small intestine, causing severe iron-deficiency anemia in humans (1).

Pre-clinical hookworm vaccine studies have focused on identifying vaccine antigens from either the dog hookworm *A. caninum, A. ceylanicum*-golden hamster, or a laboratory strain of *N. americanus* adapted to golden hamsters (46). For example, Miller et al. developed the first hookworm vaccine using whole irradiated *A. caninum* L3 larvae antigens (200). Dogs immunized with the vaccine candidate showed between 37 and 90% protection depending on the route of administration (200, 201). As a result, this vaccine was commercialized in the United States in 1973 for canines. However, it was withdrawn after 2 years because of the high cost, storage, stability, and the lack of sterilizing immunity (202).

The human hookworm vaccine initiative of the Sabin Product Development Partnership has been directed toward identifying a hookworm vaccine (49, 203). Significant efforts have been made in identifying vaccine antigens from the infective larval L3 stages as they play critical roles in host invasion, modulation of host immunity and parasite establishment (203, 204). Several L3 proteins, especially enzymes, showed promising results as recombinant vaccines in different animals, and expression systems, including the tissue invasion-related Astacin-like metalloprotease (Ac-MTP-1) (205–208), Ac-16 (209), and the two *Ancylostoma* secreted proteins (ASP-1 and ASP-2) (206, 210–212). Of these, ASP-2 was considered a lead hookworm vaccine candidate as it showed the most promising results in animal models and pre-clinical vaccine trials (206, 213, 214). For example, Bethony et al. showed that laboratory dogs immunized with recombinant ASP-2 formulated with the GlaxoSmithKline Adjuvant (AS03) significantly reduced worm burdens and clinical pathology and induced strong antibody titers compared to control animals (204). Furthermore, the sera obtained from the immunized dogs significantly inhibited the migration of L3 through tissue *in vitro* compared to sera from control dogs, suggesting that antibodies might play a critical role in protection by decreasing the number of L3 that reach the gastrointestinal tract (204). Rats immunized with Na-ASP-2 formulated with Alhydrogel also induced strong antibody response (IgG1, IgG2a, and IgM) and induced a Th2 skewed immune response (215, 216). However, after offering so much promise, the rNa-ASP-2/Alhydrogel vaccine was halted in 2008, having reached Phase I clinical trials, due to generalized urticarial reactions characterized by a high prevalence of IgE antibodies to larval antigens in individuals previously infected with or exposed to *N. americanus* in endemic populations (202, 203, 210). Thus, the mechanisms of protection from IgE-mediated disorders, typically associated with helminth infection, seemed unable to protect adults living in hookworm endemic areas from developing allergic reactions after immunization with Na-ASP-2. It was hypothesized that the induced immediate-type hypersensitivity was due to the intrinsic structural or biological properties of the Na-ASP-2 molecule (217, 218).

#### The Identification of Aspartic Protease-Hemoglobinase (Na-APR-1) and Glutathione S-Transferase-1 (Na-GST-1) as Lead Antigens

The failure of Phase I clinical trial focused attention back on to antigen selection and led scientists toward identifying vaccine antigens that were less likely to be recognized by IgE antibodies induced by the natural infection (49). Strategies included examining IgE responses in the sera from populations in countries with endemic STH infections, mutating the antigenic epitopes recognized by host IgE and using bioinformatics tools that can screen for allergenicity (212, 219). Adult hookworms suck blood from damaged vessels in the gut mucosa and digest hemoglobin using haemoglobinases (220, 221). Since neutralization of these critical enzymes would result in starvation of the parasites, leading to parasite death, these antigens were selected for the development of hookworm vaccines (49, 222). Two promising vaccines derived from the adult stage parasite were identified, aspartic protease-hemoglobinase (Na-APR-1) and glutathione S-transferase-1 (Na-GST-1) (203, 222, 223). APR is an enzyme that helps digest hemoglobin (221, 224), whereas GST is essential for parasite survival and heme detoxification (Blood-feeding pathway) (48, 225).

In pre-clinical testing, recombinant Na-APR-1 induced neutralizing antibodies (IgG1 and IgG2) against the hookworm haemoglobinase and resulted in significantly reduced blood loss, adult parasite burdens, and fecal egg counts in immunized dogs when challenged with hookworm larval (222, 224). Likewise, hamsters immunized with Ac-APR-1 showed a high level of protection (226). Importantly, IgE from individuals with hookworm infections did not recognize Na-APR-1 (224). Developing APR further as a lead antigen, Skwarczynski et al. developed an epitope-based subunit vaccine based on the A_291_Y B-cell epitope identified from the Na-APR-1, incorporated into a self-adjuvant system (Lipid Core Peptide). Interestingly, the vaccine candidate induced potent enzyme-neutralizing antibodies in mice (227). However, this study did not assess the protective immune response *in vivo*. Pearson et al. also explored a multi-antigen peptide-based vaccine against schistosomiasis and hookworm containing A_291_Y peptide from Na-APR-1, a *S. mansoni* Sm-tetraspanin-2 and Na-GST-1 antigens (228). A more recent study also showed that mice immunized with a lipopeptide-based vaccine consisting of a B-cell epitope (p3) derived from the Na-APR-1 and attached to a T-helper epitope (p25) induced a strong humoral immune response and resulted in >98% reduction in worm and egg burden following challenge infection with the rodent model hookworm, *Nippostrongylus brasiliensis* (229). Further, the same vaccine nanoparticle, when incorporated into natural and unnatural hydrophobic amino acids, also significantly reduced both worm and egg burden in orally vaccinated mice following *N. brasiliensis* challenge without the need for adjuvant (93). Pre-clinical studies also suggested that GSTs from *N. americanus, A. caninum*, or *A. ceylanicum* to be promising vaccine candidates (230). For example, Na-GSTs from the dog hookworm *A. caninum* (Ac-GST-1) elicited a significant reduction in adult hookworm burdens following challenge infection compared to control animals (225, 231).

On the basis of these and other pre-clinical data, the two lead vaccine candidates (Ac-GST-1 and Ac-APR-1) formulated individually with Th2 adjuvant Alhydrogel and TLR4 agonist (GLA) are in Phase 1 trials in the United States, Brazil, and Africa (48, 232, 233). Co-administration of both vaccines is also undergoing a clinical trial in Gabon (234).

#### Other Hookworm Vaccine Candidates

Promising results have also been achieved in hamsters immunized with DNA-based vaccines encoding the *A. ceylanicum* metalloprotease 6 (Ace-MEP-6) (63) or Ace-MEP-7 as an alternative strategy to recombinant protein production (62). Both vaccines induced significant reductions in worm burden. Additionally, a 78% egg count reduction was observed in hamsters immunized with Ace-MEP-7 (62).

Recent genomic and transcriptomic analysis of all three species of hookworms (26, 29, 30) have also helped to identify additional vaccine candidates including the two intestinally-enriched, putatively secreted, cathepsin B cysteine proteases (AceyCP1, AceyCPL) and the Kunitz-type protease inhibitor (AceySKPI3) (29). Vaccination of hamsters with AceyCP1/Alhydrogel induced a high level of protection associated with the production of high levels of antigen-specific antibodies (IgG). These antibodies also reduced the motility of the adult worms *in vitro* and induced Th2 responses (IL-4, IL-5, and IL-13) in re-stimulated splenocytes (235). Mechanistically, vaccinated animals were thought to be protected as a result of antibodies that neutralized the catalytic activity of the hookworm antigens in the gut (224, 236), although the full mechanism through which protection is conferred remains unclear. This study and others proved that parasite-secreted cysteine proteases involved in parasite nutrition are valid targets for the development of anti-parasitic vaccines (236). Table 1 summarizes candidate hookworm vaccine antigens.

**Table 1:** Major hookworm vaccine candidates.

**Antigen**	**Parasite spp**.	**Adjuvant**	**Animal model**	**Vaccine type**	**Protection %**	**References**
Glutathione-S transferase 1 (GST-1)	*A. caninum* (Ac-GST-1)	Alhydrogel	Mesocricetus auratus golden hamsters	r-protein	50.6% reduction in worm burden	(226)
Aspartic protease 1 (APR-1)	*N. americanus* (Na-APR-1)	Alhydrogel and a CpG	Dogs	r-protein	ND 66.6% reduction in egg count	(224)
	*A. caninum* (Ac-APR-1)	AS03	Dogs	r-protein	33% reduction in worm burden	(222)
	*A. caninum* (Ac-APR-1)	Alhydrogel	Mesocricetus auratus golden hamsters	r-protein	44.4% reduction in worm burden	(226)
Metalloprotease 6 and 7 (MEP-6 and MEP-7)	*A. ceylanicum* (AceyMEP-6)	ND	Syrian golden hamsters	DNA-based	80% reduction in worm burden	(63)
	*A. ceylanicum* (AceyMEP-7)	ND	Syrian golden hamsters	DNA-based	50% reduction in worm burden 78% reduction in egg count	(62)
Cysteine proteases 1 and 2 (CP-1 and CP-2)	*A*. *ceylanicum* (AceyCP1)	Alhydrogel	Syrian golden hamsters	r-protein	40–54% reduction in worm burden 54–60% reduction in egg count	(235)
	*N. americanus* (Na-CP-2)	Freund	Mesocricetus auratus golden hamsters	r-protein	29.3% reduction in worm burden	(226)

*ND, Not done; r-protein, Recombinant protein*.

In the context of human hookworm vaccine development, the existence of a human model system for the testing of hookworm vaccines is a significant advantage. Thus, controlled human hookworm infections will likely improve the early stages of vaccine efficacy testing (50).

### Experimental *Ascaris* Vaccine Candidates

Ascariasis caused by *Ascaris lumbricoides*, or *Ascaris suum* remains the most prevalent NTD worldwide. Indeed, the use of *Ascaris* egg-contaminated sewage sludge in agriculture has meant that, even in Europe, exposure to *Ascaris* is surprisingly common (237–239). *A. lumbricoides* infects around 819 million people worldwide, with children especially susceptible (240). *A. suum*, the pig roundworm, causes serious economic losses in meat-producing livestock species worldwide (241). Both parasites have a cosmopolitan distribution, an identical life cycle and are morphologically, genetically, and antigenically very similar (242–245). Consequently, efforts have been made in the past to identify vaccine antigens derived from the larval stages and ultraviolet irradiated attenuated embryonated eggs of the pig roundworm *A. suum* (246). However, the large size of the pigs, the cost and the complexities of animal husbandry, has driven many researchers to use mice and other rodent hosts as a convenient alternative for purposes of vaccine development (247). However, it is important to note that there is no rodent model that enables the *Ascaris* parasite to complete its life cycle.

Over the last decade, efforts have been made to identify crude extracts (248), recombinant proteins (249, 250), defined native molecules (251) and extracellular vesicles (252) as potential *Ascaris* vaccine candidates. These include the 66 kDa gut antigenic protein, the most immunodominant protein identified from homogenized *A. suum* adult worm fractions (251), but which was never tested *in vivo* for immunoprophylactic purposes (251). Other studies have explored targeting nematode haemoglobins as they can break down nitric oxide and hydrogen peroxide, thus potentially providing protection against innate host defenses (253, 254). However, immunizing pigs with *A. suum* hemoglobin (AsHb) in combination with QuilA adjuvant, failed to induce protective immunity following *A. suum* egg challenge (255).

Further, targeting nematode haemoglobins may offer cross species protection. For example, passive immunization of mice with a monoclonal antibody (48Eg) against hemoglobin of the nematode *Anisakis pegreffii* prior to infection with *Nippostrongylus brasiliensis* (rodent hookworm) enhanced protective Th2 immunity and significantly reduced worm burdens (256). This study also showed that 48Eg cross-reacts with the hemoglobin of several nematodes including *A. lumbricoides, N. brasiliensis, Anisakis pegreffii, Pseudoterranova decipiens*, and *Contracaecum* spp.

The public availability of the genome, and gene expression data for both *A. lumbricoides* and *A. suum* (28, 243, 245), also helped in identifying *Ascaris* vaccine peptides based on the RV approach. Indeed a recent study identified CD4 Th cell epitopes in *A. suum* ES products based on *in vitro* antigen processing and quantitative proteomic tools (257). However, the selected epitopes have yet to be tested *in vitro* or *in vivo* for their ability to induce immune responses.

To date the five major *A. suum* immunodominant antigens tested as possible vaccine candidates are; *A. suum* 16-kilodalton As16 (249), As14 (258), As24 (259), As37 (260), and As-Enol (enolase) (247). As14 and As16, identified from the sera of infected mice with *A. suum*, are localized in both larval and adult stages, as well as in the ES products of both the human and pig roundworms (23) and are homologous to the *A. ceylanicum* (Ac-16) vaccine candidate (209). Intranasal immunization of mice with recombinant rAs14 (258) and rAs16 (249) expressed in *E. coli* and coupled with cholera toxin B subunit (CTB), produced significant protection (64 and 58% respectively), compared to non-vaccinated mice following *A. suum* infection. Furthermore, recombinant rAs16 induced a 58% reduction in the recovery of the lung-stage in a pig animal model, associated with high levels of IL-4 and IL-10 and high titers of rAs16-specific mucosal IgA and serum IgG antibody (261). Through the use of sera from rAs16-CTB immunized mice, As16 was localized to the worm hypodermis and intestine (249, 261). Sera from rAs16-CTB immunized mice was also shown to inhibit molting of *A. suum* L3 *in vitro* (261). Interestingly, subcutaneous vaccination of mice with yeast-expressed As16 formulated with Montanide ISA720 adjuvant significantly reduced larval recovery and induced a Th2 immune response against challenge infection. In contrast mice immunized with rAs14 formulated with ISA720 failed to induce protection (262). Other studies have used plants as alternative, attractive, vaccine producing factories. For example, mice fed with As16-transgenic rice fused with CTB showed a significant reduction in the number of larvae following challenge infection (263).

Promising results were seen in mice immunized with the nematode-specific protein (As24) expressed in *E. coli* and emulsified with Freund's Complete Adjuvant (FCA), although the potential for translating these studies to man is limited by the choice of adjuvant. The vaccine resulted in a 58% reduction in lung larval burden post-challenge and induced a Th1/Th2-mixed type immune response characterized by elevated levels of IgG, IFN-γ and IL-10 (264). As24 homologous proteins were also identified in *Ascaris lumbricoides* and the dog parasitic nematode *Toxocara canis* (259). Anti-As24 IgG also inhibited molting of *A. suum* lung stage, suggesting As24 plays a critical role in the development of *Ascaris* larvae (259). Further, the protective immune response to *A. suum* larvae correlated with the induction of IgG1 and IgM, and not with IgG2 in pigs immunized with As14, and As24 fractions from adult worms (265, 266).

It has also been proposed that As37, a member of the immunoglobulin superfamily, identified from the adult and larval stages as well as in the hypodermis and muscle of *A. suum* is a potential *Ascaris* vaccine candidate (260, 267). Mice immunized with rAs37 formulated with AddaVax adjuvant induced a significant 49.7% larval worm reduction after challenge infection compared to control animals. Protection was associated with the production of high levels of serological IgG1 and IgG2a and stimulated the production of IL-4, IL5, IL-10, and IL-13 cytokines, suggesting that both Th1 and Th2 immune responses are essential for worm expulsion (250). Interestingly, the AddaVax adjuvant performed better than the Th1 adjuvant MPLA and the Th2 adjuvant Alhydrogel. Remarkably, sequence analysis revealed that As37 is highly conserved in other STHs including *N. americanus, A. ceylanicum, A. caninum*, and *T. muris*, but not in humans, suggesting that the nematode-conserved antigen could serve as a pan-helminth vaccine antigen (250).

The inorganic *A. suum* pyrophosphatase is another promising vaccine candidate expressed throughout the life cycle and localized in the surface and adult reproductive tissues (268). Knockdown of *A. suum* pyrophosphatase by RNAi has indicated the importance of this phosphatase in larval development and molting (269). Furthermore, immunization of mice with rAs-PPase expressed in *E. coli*, and formulated with TiterMax Gold adjuvant resulted in >70% protection against the L3 stage, drove a high serum IgG1 response, and significant production of splenic IL-10 (270, 271).

Moreover, enolase (As-Enol-1), found in the *A. suum* larva, adult ES and EVs has been shown to play a critical role in larval development (272, 273), and triggering *in vitro* macrophage nitric oxide production (274). Vaccination of Kunming mice with recombinant As-Enol-1 leads to a 61.13% reduction (*P* < 0.05) in larval recovery and elicits a Th1/Th2 (IFN-γ, IL-2, IL-4, and IL-10) immune response (247).

Gazzinelli-Guimaraes et al. (248) also evaluated the immunological, potential clinical impact and protective immune responses of three different *Ascaris* extract vaccines formulated with the MPLA adjuvant. Mice immunized with crude extract of adult worm (ExAD) exhibited a significant reduction (51%) in the total number of migrating larvae recovered in the lung tissue and bronchoalveolar lavage; crude extract of adult worm cuticle (CUT) 59%, and crude extract of infective larvae (L3) (ExL3) 61% compared to the non-immunized mice. Protection was associated with a marked systemic production of *Ascaris*-specific IgG1 and IgG3 subclasses and a significant increase in systemic IL-5 and IL-10 (pre-challenge) and lung IL-10 (post-challenge). ExL3 and CUT protection was also associated with less tissue damage and pulmonary tissue inflammation as well as reduced pulmonary dysfunction following *Ascaris* challenge. Furthermore, the passive transfer of purified antigen-specific IgG antibodies from mice immunized with ExL3, CUT, and ExAD into naïve mice induced a significant reduction in parasite burdens in lungs of 65, 64, and 64%, respectively (248). These results suggest that vaccine induced antibodies play a crucial role in reducing larval migration and subsequent larval burden in the lungs.

Overall, the protective anti-*Ascaris* immunity (Table 2) observed in all the experimental animal model points toward a Th2-biased immune response, associated with the production of high levels of parasite-specific IgG1. Analyses of vaccine-driven immunity has suggested more mixed Th1/Th2 immune responses may be at play (250); however, over robust Th1-type responses are counter protective. Again, as with hookworm, the immunological mechanisms underpinning vaccine induced immunity to *Ascaris* infections are only partially understood.

**Table 2:** Major Ascaris vaccine candidates.

**Antigen**	**Vaccine type**	**Protection % (Reduction in lung larval burden)**	**References**
As14	r-protein	64%	(258, 266)
As16	r-protein	58%	(249, 261, 262)
As24	r-protein	58%	(259, 264)
As37	r-protein	69%	(275)
As42	r-protein	67%	(266)
Enol-1	DNA	61%	(247)
As66k	ND	ND	(251)
• Crude extract of adult worm (ExAD) • Crude extract of adult worm cuticle (CUT) • Crude extract of infective larvae (L3) (ExL3)	Crude extract	• ExAD 51% (*p* < 0.01) • CUT 59% (*p* < 0.001) • ExL3 61% (*p* < 0.001)	(248)

*ND, Not done; r-protein, Recombinant protein*.

### Experimental *Trichuris* Vaccine Candidates

Historically, the global health community have focused their vaccine research on hookworm and ascariasis and somewhat neglected trichuriasis, despite the fact that trichuriasis is the second most common STH infection after ascariasis (13). Around 477 million people are estimated to be infected with *Trichuris* infection, with the highest intensity of infection seen in school-aged children (1, 2).

One of the first attempts at identifying a non-living vaccine for *T. trichiura* was conducted in the mouse model *T. muris*, using adult and larval worm somatic antigens to stimulate protective immunity in infected animals (278). Vaccination of mice with *T. muris* somatic antigens that were isolated from NIH mice and emulsified in Freund's incomplete adjuvant stimulated protective immunity and a 92% reduction in worm burden after infection (278). Wakelin and Selby (278) also demonstrated that soluble antigens from the anterior region of adult worms were more effective in stimulating immunity than antigens prepared from the posterior region. *T. muris* adult worm homogenate and ES products from both adult and larval stages have been used in early-stage vaccine development (279, 280). Route of antigen delivery has also been explored. For example, a level of protection can be achieved against *T. muris* using homogenized adult antigens combined with cholera toxin as an oral vaccine (281). However, a 100% reduction in worm burden can be achieved through subcutaneous (s.c.) vaccination with adult antigens emulsified in Freund's complete adjuvant (281). Moving to slightly less crude antigen preparations, but also exploring the route of administration, Jenkins and Wakelin (279) showed that vaccinating mice subcutaneously with 100 μg of the excreted and secreted ES products of adult *T. muris* parasites without adjuvant, was more effective than intraperitoneal vaccination, also in the absence of adjuvant. More recently, Dixon et al. (85) showed that subcutaneous vaccination of naturally susceptible AKR mice with *T. muris* ES emulsified with Freund's incomplete adjuvant (IFA) induced expulsion of a high-dose infection. This study also described priming of the immune response to subcutaneous vaccination as occurring in peripheral lymph nodes draining the site of vaccination, that the ensuing protective immune response was of a mixed Th1/Th2 type and eluded to the effector mechanisms involving some form of intestinal antibody-mediated cellular immune response (85).

Interest in identifying host protective material in the extracellular vesicle (EV) components within helminth secretions has increased recently (282–284). For example, Shears et al. (276, 285) showed that vaccinating C57BL/6 mice with either ES fractions or EVs isolated from *T. muris* without an adjuvant and prior to infection with a low-dose of *T. muris* eggs significantly reduced the worm burden compared to the PBS-injected group, inducing a mixed Th1/Th2 response. Vaccination of mice with ES fractions stimulated long-lasting protection against chronic infection characterized by the production of high levels of IL-9 and IL-13. However, the sera from ES vaccinated mice did not protect naïve mice from *T. muris* chronic infection, suggesting that anti-parasite antibodies did not play a critical role in protection (285). Shears et al. also demonstrated that vaccination with EVs boosted IgG1 antibody production against *T. muris* ES proteins, suggesting that there is extensive overlap in protein content between the EVs and ES (276).

Gomez-Samblas et al. (277) have also identified a vaccine candidate that potentially could work against a variety of helminth parasites, including *T. muris*. This candidate is based on the recombinant serine/threonine phosphatase 2A from the nematode *Angiostrongylus costaricensis* (rPP2A), formulated as a lipopeptide and conjugated with a self-adjuvant oleic-vinyl sulfone (OVS). Interestingly, intranasal immunization of AKR mice with the vaccine candidate prior to *T. muris* challenge led to a marked reduction in the number of adult parasites. The immunized mice also developed a combined Th17/Th9 response orchestrated by the cytokines IL-25, IL-17, and IL-9 (277). The same vaccine candidate has also been tested in lambs and was found to provide significant protection against the ovine helminth *Haemonchus contortus* and *Teladorsagia circumcincta* infection (286).

Briggs et al. (91) had developed two vaccines against trichuriasis based on *T. muris* whey acidic protein (rTm-WAP49) and *T. muris* WAP fragment fusion protein (rTm-WAP-F8+Na-GST-1) formulated with Montanide ISA 720 adjuvant. Vaccinating AKR mice with the vaccine candidates three times at 2-week intervals prior to *T. muris* challenge induced a partial reduction in worm burden (48 and 38%, respectively) (91). The authors also showed that both humoral and cellular immune responses were induced and characterized by elevated antigen-specific IgG1 and IgG2c antibodies and Th2 (IL-4, IL-9, and IL-13) cytokines in the draining inguinal LNs, draining mesenteric LNs and spleens of vaccinated mice (91).

Despite these promising results (Table 3), subunit vaccines often require substantial adjuvant and often do not provide sufficient protective cellular immunity compared with other vaccine approaches (76). Thus, a recent study identified a promising vaccine candidate based on major histocompatibility complex class II (MHC-II) T-cell epitopes identified from the whole genome of the *Trichuris*, incorporated into Hepatitis B core antigen VLP (79). The four epitopes were identified from chitin-binding domain-containing proteins and chymotrypsin-like serine proteases. *In vitro* studies showed that the VLPs were internalized and co-localized in the antigen-presenting cells (dendritic cells and macrophages) lysosomes and stimulated the production of pro-inflammatory and anti-inflammatory cytokines. Remarkably, immunization of mice with four VLPs expressing *Trichuris* T-cell epitopes induced a significant reduction in worm burden following challenge infection compared to control animals without the need for an adjuvant. Protection was associated with the induction of a mixed Th1/Th2 immune response characterized by the production of *Trichuris*-specific IgM and IgG2c and the production of mesenteric lymph node-derived Th2 cytokines and goblet cell hyperplasia.

**Table 3:** Major Trichuris vaccine candidates.

**Antigen/adjuvant**	**Vaccine type**	**Protection % (Reduction in worm burden)**	**References**
ES/FIA	Crude	97%	(85)
EVs and EVs fractions	Crude	Significant reduction (% was not indicated)	(276)
rPP2A/OVS	r-protein	97.90%	(277)
• rTm-WAP49/Montanide ISA 720 • rTm-WAP-F8+Na-GST-1/Montanide ISA 720	r-protein	• 48% • 38%	(91)
VLPs expressing *Trichuris* T-cell epitopes	Epitope-based	50%	(79)

*OVS, Oleic-vinyl sulfone; FIA, Freund's incomplete adjuvant*.

## Conclusion and Lessons Learned

STH infections are common in the world's poorest people living in low- and middle-income countries and are linked to detrimental effects on maternal and child health. Several studies have been conducted over the years to develop vaccines as cost-effective methods to control STHs infection (49). However, the process of vaccines development against STH parasites is complicated and faces several challenges. Thus, multiple broad-ranging factors have to be taken in to account when developing a protective vaccine to meet the immunogenicity, safety, and efficacy criteria of regulatory institutes such as the US Food and Drug Administration. These include identifying protective antigens that do not trigger unwanted allergic-type immune responses, the best route of administration, the vaccine type and adjuvant to be used and how to elicit protective immunity in the face of regulated immune environments typical of chronic STH infections (287). Underpinning all these factors is a need to understand the immune response induced by STHs and the nature of vaccine-driven protective immune responses, such that one can strategically design vaccines to drive the right sort of quality of immune response in susceptible hosts. There is a significant problem in vaccinology in that subunit vaccines, which are composed of antigenic proteins, are often poorly immunogenic and fail to stimulate memory immune responses. Furthermore, there is a knowledge gap in understanding the mechanisms of action of vaccines and how they stimulate the required T- and B-cell responses. Targeting of antigen to specific antigen-presenting cell subsets is a promising strategy going forward. Coupling of a monoclonal antibody to antigen poses technical challenges, requiring fusion protein engineering or cross-linking, both of which can fail or produce low yields. However, overcoming these technical barriers is likely to be far-reaching in the context of STHs, where vaccine delivery is likely to occur in the context of pre-existing chronic infections. Despite an increase in STH vaccine research, it remains a disappointing truth that no human anti-STH vaccine currently exists. Among the lessons learned over the last decade is the importance of gaining international pharma company attention and support in order to bring antihelminth vaccines to trials. Further, raising public health awareness of the enormous threat presented by STH infections to the economy and health of at-risk populations, including their impact on susceptibility to other pathogens (49, 192) is critical. This has never been more relevant than now, given today's climate of emerging infectious diseases including SARS-CoV-2.

## Author Contributions

AZ researched the literature, drafted the manuscript, and compiled the tables. KE edited the manuscript and revised the content. All authors contributed to the article and approved the submitted version.

## Conflict of Interest

The authors declare that the research was conducted in the absence of any commercial or financial relationships that could be construed as a potential conflict of interest.
